# What aspects of health and wellbeing are most important to parent carers of children with disabilities?

**DOI:** 10.1111/hex.14085

**Published:** 2024-06-06

**Authors:** Caomhan McGlinchey, Phillip Harniess, Aleksandra J. Borek, Alice Garrood, Annabel McDonald, Fleur Boyle, Stuart Logan, Christopher Morris

**Affiliations:** ^1^ Peninsula Childhood Disability Research Unit (PenCRU) and NIHR Applied Research Collaboration South West Peninsula (PenARC) University of Exeter Medical School, University of Exeter Exeter UK; ^2^ Nuffield Department of Primary Care Health Sciences, Medical Sciences Division Radcliffe Observatory Quarter, University of Oxford Oxford UK

**Keywords:** health, mental health, outcomes, parent carers, parents, wellbeing

## Abstract

**Introduction:**

Parent carers of children with special educational needs or disabilities are at risk of poorer health and wellbeing outcomes because of the distinct and challenging circumstances they face. Evaluations of interventions promoting the health of parent carers should focus on measuring the aspects of health and wellbeing which are most relevant to this group. As part of a programme of research on parent carer‐focused interventions, this study aimed to understand which aspects of health and wellbeing are perceived by parent carers as most meaningful and important.

**Methods:**

A qualitative study using semistructured online interviews was conducted. A purposive sample of parent carers was interviewed about relevant health and wellbeing outcomes. Transcripts were analysed thematically.

**Results:**

Thirty parent carers were interviewed, 19 of whom had experienced a health‐promoting intervention, either as participants (*n* = 14) or facilitators (*n* = 5). Three main themes were identified: ‘self, identity and beliefs’; ‘social connections and support’ and ‘health‐promoting practices and outcomes.’ Each theme encompassed the challenges participants faced, and the changes that helped them overcome these challenges. ‘Self‐identity’ challenges focused on the overwhelming nature of the parental care role and the emotional impact of this. Changes were brought about by developing a positive mindset, increasing confidence, and reconnecting with aspects of their identity which were important to them before they became parent carers. Challenges related to ‘social connections’ reflected parent carers' isolation. Change was brought about through increased peer support and peer interactions. Parent carers experienced challenges in terms of ‘health‐promoting activities’ because they lacked free time and experienced poor physical health. Changes were brought about by engagement in health‐promoting activities of various kinds.

**Conclusion:**

Parent carers view health and wellbeing in terms of overcoming the common challenges they face as a group. These challenges reflect the ways in which their physiological and psychological needs are often unmet. Researchers interested in measuring parent carer health and wellbeing should consider the specific challenges this group face, as well as theoretical frameworks which can make sense of these challenges, such as self‐determination theory.

**Patient or Public Contribution:**

Our team carries out patient and public involvement (PPI) through a Family Faculty group facilitated by a Family Involvement Co‐ordinator (A. McD.) who is herself a parent carer. A study‐specific PPI working group was established which included members of the Family Faculty. The PPI group advised on various aspects of the research as reported in the paper. The manuscript was co‐authored by the team's Family Involvement Co‐ordinator (A. McD.).

## 1 INTRODUCTION

Parent carers of children with special educational needs or disabilities (SEND) are at increased risk of poor health and wellbeing outcomes.^1^, ^2^, ^3^, ^4^, ^5^, ^6^, ^7^ They feel less able to pursue their interests than other parents do,^8^ and they are more likely to withdraw from work.^9^, ^10^ They also engage in fewer social relationships outside of their immediate family.^11^ For parent carers, poorer outcomes have been associated with financial concerns, stress and social isolation.^4^, ^5^, ^6^, ^7^, ^12^, ^13^ Population‐based studies, meanwhile, indicate that parent carers' health problems are long‐lasting, and often worsen over time.^4^, ^5^, ^6^, ^7^, ^12^, ^13^

There are a growing number of parent carer‐focused interventions seeking to support aspects of parent carers' health and wellbeing.^14^, ^15^, ^16^, ^17^, ^18^, ^19^ These interventions aim to: teach parent carers about managing specific conditions and/or disabilities; share parenting skills and/or therapeutic approaches; empower parents in their interactions with professionals; and/or promote health and manage stress. Parent carer‐focused interventions are often delivered in groups. Trainers or facilitators may be parent carer peers and/or professionals, and they often deliver their interventions in pairs. Some programmes have been targeted exclusively at mothers or fathers, while others have focused on all parent carers, or even entire families.

The Healthy Parent Carers (HPC) programme is a co‐created, community‐based behaviour change intervention, designed to promote positive health and wellbeing among parent carers.^12^, ^13^, ^15^, ^20^ It is designed and delivered by parent carers, and aims to improve the health and wellbeing of parent carers by encouraging groups of parent carers to engage in evidence‐based health‐promoting behaviours. The programme is delivered to groups either face‐to‐face or online and involves a series of daytime or evening weekly sessions delivered by two trained facilitators who are parent carers themselves. There are twelve modules that aim to increase parent carers' engagement in health‐promoting behaviours (‘CLANGERS’: Connect, keep Learning, be Active, Notice, Give, Eat Well, Relax and Sleep).^15^, ^21^, ^22^

Following a feasibility randomised controlled trial of the HPC programme,^12^ qualitative analysis demonstrated that HPC was perceived by participants to be beneficial and impactful.^20^ However, the analysis of the distribution of responses to items in the patient‐reported outcome measures, Patient Health Questionnaire‐9, Warwick‐Edinburgh Mental Wellbeing Scale and EuroQol‐5D,^23^, ^24^, ^25^, ^26^ indicated a potential lack of sensitivity to the benefits articulated by participants. This suggested that parent carers may have a particular experience of health and wellbeing that is not easily captured using items in existing generic or dimension‐specific measures, warranting further investigation.

A recent systematic review of self‐report measures identified 99 questionnaires measuring 196 dimensions of wellbeing.^27^ The authors of that review recommended that researchers carefully consider the wellbeing domains which are of interest and most relevant to their investigation. As part of a broader programme of research focused on parent carer interventions, we sought in this study to understand which aspects of health and wellbeing are perceived by parent carers as the most meaningful and important. The results will assist future researchers when determining which outcomes to measure.

## 2 METHODS

This qualitative descriptive study consisted of semistructured online interviews drawing on rapid qualitative research techniques.^28^ Reflexive thematic analysis was used,^29^, ^30^ and an interpretivist epistemological perspective was taken. We considered that data are constructed via multiple perspectives, allowing us to build connecting themes from shared participant experience.^31^

### 2.1 Patient and public involvement (PPI)

Our team carried out PPI through a Family Faculty group facilitated by a Family Involvement Co‐ordinator (A. McD.) who is herself a parent carer. The Family Faculty is made up of families containing children with disabilities who are living in the United Kingdom, and who are interested in the work being carried out by the Peninsula Childhood Disability Research Unit.^22^, ^32^ Members are invited to get involved in our research in a number of ways.^33^ For example, they are encouraged to suggest research questions, get involved in project working groups, and make suggestions about the language we use—including helping us to design plain language summaries. For this study, we convened a study‐specific PPI working group who were volunteers from members of the Family Faculty. The PPI group advised on various aspects of the research. The Guidance for Reporting Involvement of Patients and the Public, Version 2 short form is used to describe the collaboration with our PPI group for this study more comprehensively (Table 1).^34^

**Table 1:** Public and Patient Involvement (PPI) in this research described using the Guidance for Reporting Involvement of Patients and the Public, Version 2 short form.

Topic	Item
Aims	To ensure that the research remained focused on the parent carer perspective in addressing its main aims. To advise on all aspects of the study methods, review emerging findings and interpret the results.
Methods	Eight parent carers are involved, seven of whom have previously participated in the Healthy Parent Carer (HPC) programme as participants (six), facilitators (one); one member of the PPI group has not participated in HPC but is familiar with the programme. Group meetings are co‐designed and facilitated between two researchers and the Family Involvement Coordinator (who is a parent carer) to support input from parent carers relating to the study aims design, including sampling and recruitment strategies, and analyses. Six group online meetings have been convened to date using Zoom; two one‐on‐one meetings have been arranged separately on two occasions. As data began to be collected, emerging analysis was shared with the group for discussion through vignettes, to sense‐check and challenge the assumptions of the researchers to build a more robust representation of the data. PPI members were also involved outside of the group meetings reviewing study documents such as the PIS, study adverts, and consent form.
Results	PPI is contributing to the study in several ways: Setting study priorities. Advice on sampling and recruitment strategies. Development of the study topic guide through pilot group interviews and cognitive testing. Critical analytical development, where parent carers in the PPI group were invited to challenge and build on emerging analysis and coding framework. This resulted in placing greater emphasis on the effect of the parent carer identity, social isolation, comparison and confidence on health and wellbeing outcomes, alongside stress, sleep deprivation and low mood. Dissemination of findings; the PPI group will help co‐produce plain English summaries.
Discussion and conclusions	PPI is highly influential in multiple areas as described. In particular, the early analytical framework was clarified surrounding the core challenges parent carers face relating to health and wellbeing. Greater emphasis and relevance were placed on the influence of the parent carer identity and confidence, as this resonated strongly with the PPI group through in‐depth discussion.
Reflections/critical perspective	The study is utilising rapid qualitative techniques which potentially impacts upon the extent to which the PPI group can influence analysis. Nevertheless, the PPI group are very keen to remain engaged to ensure that all analyses are discussed in sufficient depth insofar as possible.

### 2.2 Participants

Inclusion criteria for this study were parent carers of children with SEND living in England. Initially, parent carers who had been involved in the HPC programme were focussed on for recruitment. Of these parent carers, 70 indicated that they were willing to be approached for research purposes, 59 of whom had been participants on the programme, and 11 of whom had been facilitators. This group was invited to participate by email. Potential interviewees were also invited via the HPC website and social media, further snowballing via the PPI group and an email list of approximately 200 parents of children with disabilities who had previously indicated that they would be interested in research. Study adverts were written in plain English, and included the research title, research questions, and researchers' contact details. Adverts included a link to the study website, where potential participants could find more information about the study, and a link to an online form where they could register their interest.

Those who had expressed their interest were contacted by telephone or e‐mail and asked to provide demographic data, including their name, gender, telephone number, level of education and ethnicity. Participants were also asked for their postcode to include an index of multiple deprivation data using the English Indices of Multiple Deprivation 2019.^35^ Registrants' details were downloaded onto a spreadsheet and screened for eligibility. Eligible registrants were again contacted by telephone or e‐mail to complete a consent form and arrange an interview date and time. After the interview, interviewees were offered a £35 voucher for taking part.

The sample size was determined pragmatically. We initially planned to recruit approximately 25 interviewees sampled across different groups to produce sufficient data to answer the research question. This meant recruiting those with knowledge of the course (facilitators and previous participants), as well as those with no experience of the course. Having recruited 25 individuals for interview, we observed that we had recruited few parents of children with physical disabilities. We, therefore, recruited an additional five parents of children with physical disabilities to fully address the physical health aspect of the research question.

### 2.3 Data collection

We conducted one‐to‐one semistructured interviews via video conference calls. Interviews were conducted by one of two researchers (C. McG./P. H.). They lasted approximately 1 h. A semistructured topic guide was created and piloted with the PPI group. It included open questions about parent carer health and wellbeing. Interviews were audio‐recorded and transcribed verbatim.

The goal of each interview was to identify meaningful and important aspects of health and wellbeing. Interviewees who had participated in the HPC programme were asked to reflect on their health and wellbeing both before and after the programme. Interviewees who had not participated in HPC were asked to reflect on the impact they would expect a programme like this to have, that is, a programme aimed at improving health and wellbeing among parent carers. All interviewees were then asked to reflect more broadly on which outcomes would be important when evaluating an intervention focused on parent carer health and wellbeing.

### 2.4 Data analysis

We drew on rapid qualitative research techniques,^28^ including a team approach to analysis, concurrent data collection and analysis, and completion of rapid summaries after interviews to facilitate prompt analysis and inform subsequent data collection. Findings were regularly reviewed with the wider research team and periodically with the study PPI group until data saturation was reached.

Verbatim transcripts of audio files were uploaded to NVivo software for coding. To develop a coding framework, three researchers independently coded a subsample of transcripts (C. McG. and P. H. *n* = 4 each, A. B. *n* = 2). Each researcher arranged the codes into candidate categories related to aspects of health and wellbeing. The categories and codes were compared and discussed with the team to find a consensus for one combined codebook. Subsequent analysis of transcripts by C. McG. and P. H. was mapped onto the codebook. Where new and relevant phenomena were observed, additional discussions saw the further development of the existing codebook. The codebook could be applied consistently across HPC facilitators, HPC participants and HPC nonparticipants, so the findings for each group are not contrasted in the results section. We applied additional rapid qualitative techniques with the final six interviews. Audio files (without full transcripts) were used to produce rapid summaries which were mapped onto the existing coding framework, with only short key segments or quotes transcribed.^28^, ^36^

The researchers conducting the analysis included two males and a female from diverse professional and academic backgrounds (educational psychology, physiotherapy and sociology). To remain cognisant of their positionality during interviews and within analysis, they used reflexive practices such as writing memos, (peer) supervision and discussing differences in coding interpretations with the team. Once data saturation had been reached, further triangulation methods were deployed which meant discussing data analysis concurrently with the PPI group to sense‐check and inform its development, in line with current guidance.^37^

## 3 RESULTS

Thirty parent carers were interviewed (Table 2); 14 had been participants in the HPC programme, whereas 5 were Lead or Assistant Facilitators on the programme. Eleven were parent carers who had no prior connection to the HPC programme.

**Table 2:** Characteristics of interviewed parent carers (*n* = 30).

		*N*	%
Parent carer mean age—years (range)		47 (range 33–76)	
Parent carer sex	Female	24	80.0
	Male	6	20.0
Partner at home	Yes	24	80.0
	No	6	20.0
Parent carer ethnicity	Asian, Asian British	4	13.3
	White	25	83.3
	Mixed	1	3.3
Parent education	Degree or above	18	60.0
	A‐level or equivalent (finished education 18 years old)	7	23.3
	GCSE (finished education at 16 years old)	5	16.6
Previous participation in HPC	Facilitator in HPC	5	16.7
	Participants in HPC	14	46.7
	Nonparticipants in HPC	11	36.7
Child's main diagnosis	Autism	24	58.9
	Autism with ADHD	4	9.8
	Learning disability	4	9.8
	Severe congenital neuromotor disorder	4	9.8
	Cerebral palsy	2	4.9
	Down syndrome	1	2.4
	Chronic fatigue syndrome	1	2.4
	Undiagnosed (social and communication difficulties)	1	2.4
Mean child age—years (range) (*n* = 41)		12.8 (3–28)	
Deprivation in area of residence (indices of multiple deprivation; quintiles)	1 (most deprived)	6	20.0
	2	6	20.0
	3	5	16.7
	4	7	23.3
	5 (least deprived)	6	16.7

Abbreviations: ADHD, attention deficit hyperactivity disorder; GCSE, General Certificate of Secondary Education; HPC, Healthy Parent Carers.

We developed three interconnecting themes which captured the areas of health and wellbeing most significant to our interviewees. These were: ‘self, identity and beliefs’, ‘social connections and support’ and ‘health‐promoting activities and outcomes’. Each section below begins with a description of how the challenges faced by parent carers have impacted different aspects of their health and wellbeing. We then discuss which positive changes they have experienced—or would hope to experience—as a result of an intervention targeting their health and wellbeing. We illustrate the findings with quotes, attributed with participant numbers and indicating whether they were HPC participants (HPC‐P), nonparticipants in HPC (HPC‐NP) or HPC facilitators (HPC‐F).

### 3.1 Self, identity and beliefs

We found that perceptions of parent carers' health and wellbeing were related to their social identity as parent carers and the expectations associated with this role.

Challenge: the overwhelming parent carer role

A core challenge for parent carers was the way in which they prioritised the parent carer identity over other applicable social identities. Supporting the ongoing needs and vulnerabilities of their children became the dominant focus of their daily lives, which had negative implications for their health and wellbeing because of the physical and emotional strain. However, this fact was compounded by the struggle these parents experienced finding time to pursue their own interests or addressing their own physical and mental health needs.

Change: reconnecting with and developing new self‐identities

Parents' health and wellbeing improved when the parent carer identity or ‘role’ no longer dominated their thoughts and behaviours. When they no longer thought of themselves solely as parent carers, parents described reconnecting with previously significant aspects of their identity—or even forging new social identities. As a result, they felt ‘calmer’ and made ‘decisions which [are] more constructive’.

I'm more now than just a parent or parent carer. I'm a trainee lawyer, I'm a civil servant, I'm a friend, I'm a brother, I'm a son … you can get quite fixated with one part of your life and that actually makes it more stressful. (P18, HPC‐P)

Challenge: negative affect and emotional exhaustion

Parent carers described experiencing low mood and depressive symptoms. This could result in negative affect, which could spiral and result in ‘emotional exhaustion’ or ‘burnout’. Some fathers discussed feelings of anger and withdrawal rather than of depression, but they still described feeling ‘stuck in a rut’ and being too ‘powerless to change things.’ In many cases, this negative thoughts and emotions could ‘spiral’ as a result of negative experiences—such as when parent carers had to ‘fight’ for resources from public service providers. The exhausting, stressful and time‐consuming interactions associated with this ‘fight’ demonstrated how parent carers’ choices and opportunities were constrained by their situation. This often reduced their sense of optimism.

Parent carers also described negative affect in the form of the ‘constant worry’ which they felt for the health and wellbeing of their child. Some parents compared their current lives with the lives they had envisioned before becoming parent carers, which could result in an emotionally draining grieving process.

… there's a lot of comparing—not just your children and your parenting to other people's children and their parenting—but also your reality to the reality you were prepared for. (P14, HPC‐P)

Change: perspective transformation and developing a more positive mindset

Parent carers who participated in the HPC intervention described being able to develop a more positive mindset and feeling a renewed sense of purpose in life. This sense of purpose was linked to parent carers' reconnection with aspects of their identity which had become less important since becoming parents. This included the development of new interests.

Another important process in terms of developing a more positive mindset was working through and beginning to accept circumstances that cannot be changed. Among some interviewees, acceptance was found to reduce stress and increase their joy in their child.

… in the big picture … I'm not doing a bad job. She's a fantastic kid. She's gonna be just fine. (P14, HPC‐P)

Parents described how learning about their child and their child's condition helped them to cope with negative emotions. This also helped them accept their identity as parent carers. Acceptance also helped parent carers manage day‐to‐day challenges, such as the unpredictable behaviour associated with their child's needs. Therefore, while the process of acceptance is unlikely to be linear, it was described as vital by those parents who developed a more positive outlook.

I've grown as a person over the years, I've tried to have a different thought process that we only live once. It's not worth dwelling on things and if we can do things to make it better, we can. (P23, HPC‐NP)

Challenge: low confidence and self‐esteem

Parent carers' experiences of negativity were also linked to their self‐confidence (their attitude about their own capabilities and skills) and their self‐esteem (their perception of their own value and self‐worth). Self‐esteem and confidence were each undermined during interactions with public services, where parents commonly expressed feeling ‘invalidated’ and ‘low status’. These feelings extended to their interactions with broader society, and sometimes emerged in their internal narratives as well.

… I'd got in a bit of a rut of really being negative and hard on myself. (P4, HPC‐P)

Low confidence could develop due to negative social comparisons with parents of children without disabilities. These comparisons intensified parent carers' sense of isolation from ‘mainstream’ parenthood norms and expectations. Parent carers with low confidence often described feeling misunderstood by other parents, as well as by family and friends. They often felt like they did not ‘belong’ in social situations. This could lead them to disengage from others through self‐isolating behaviours because isolation protected them from the difficult feelings associated with these interactions. However, in the longer‐term, this approach could shrink an individual's social circle, leading to increased feelings of loneliness.

Change: increased confidence and self‐care

Parent carers found that increasing self‐confidence and self‐esteem had a positive influence on their general wellbeing. This was achieved through positive social interactions, often with parent carer peers who understood their situation and provided ‘value‐free’ encouragement.

[feedback from peers] makes you value yourself and see what amazing jobs we all do and have confidence that you're doing the right thing… (P5, HPC‐P)

Improved confidence often led to more autonomous decision‐making and trying new things, such as engaging in hobbies, applying for jobs, and volunteering in the community.

I don't think I would have applied for any jobs if I hadn't done the course because I was feeling quite rubbish about myself and my confidence was really at zero. (P6, HPC‐P)

These new activities and healthy practices increased feelings of satisfaction, self‐worth, and self‐efficacy, creating a more positive feedback loop.

… it's taking ownership of those decisions that have been taken out of your hands so far. But now, you're taking back ownership and control. (P19, HPC‐F)

Many parent carers found it necessary to give themselves ‘permission’ before engaging in self‐care. However, this was more likely to occur when parent carers' self‐esteem and self‐worth had improved. Increased self‐esteem appeared to support more sustainable self‐care practices. For example, parents with higher self‐esteem were better able to effectively balance self‐care with their carer role because they could effectively establish boundaries around self‐care. For some parent carers, setting boundaries around self‐care meant advocating for themselves by saying ‘no’ to others.

Now I can just go, ‘no, actually, I can't do that. I need this time for me’. (P20, HPC‐P)

### 3.2 Social connections and support

Challenge: parent carers feel isolated

As previously described, social isolation was a common experience amongst parent carers, and this developed because of the perceived direct and indirect judgement and lack of understanding from friends, family, professionals, and the public.

… in the mainstream world, so to speak, you're a bit of an outlier … this world is like a different world I did not know existed. (P3, HPC‐P)

Parents cited a lack of time and not feeling that they had the emotional capacity to socialise with others, while dads emphasised the difficulties they had seeking social connections and support. Interviewees also described ‘quite a bit of family breakdown’ in their personal lives, where close relationships could become strained due to the pressure and stress associated with the parent carer role.

Change: group interventions reduce isolation and increase peer support

Parent carers who sought out like‐minded peers reported feeling less isolated because of a natural connection to other parent carers. On parent carer health and wellbeing programmes, parent carers reported valuing the immediate empathetic ‘short‐hand’ between group members.

I think that that's a huge thing, being able to connect with other parent carers who just get it. You haven't got to explain to them what it's like. (P5, HPC‐P)

Parent carers could also ‘sense‐check’ with one another and feel reassured that they were doing a ‘good enough’ job, increasing confidence and self‐esteem. Parent carers offered each other practical support, sharing specific knowledge about what services were available locally and what strategies they might try. Targeted peer support (e.g., through parent carer support programmes) enabled collaborative problem‐solving around familiar difficulties.

Increased confidence and self‐esteem could also lead parent carers to form healthier and more supportive social networks. This meant developing healthy relationships and ending unhealthy ones. Newly confident parent carers became more likely to seek out new connections.

… it's definitely given me a kick up the backside to go and integrate into society and give back a bit more. (P3, HPC‐P)

I didn't really realise how much better friendships and relationships with new people could be. So, I took that leap. (P6, HPC‐P)

Parents joined new groups, volunteered locally, and went into paid employment. For some, this ‘leap’ resulted in a virtuous cycle, where participants' positivity and confidence moved them away from self‐isolating patterns of behaviour, and towards establishing new connections with people. Successfully seeking out these new connections was then reported to reduce stress.

… stress was alleviated by these connections and having a purpose other than caring for my family. (P6, HPC‐P)

Confident parents were also better able to ‘operationalise’ their existing support network, meaning they would ask friends and family for support when needed. The virtuous cycle continued because parent carers had more time for further health‐promoting activities—such as self‐care.

… in order to improve your health and wellbeing … you can't do it on your own, you have to have your network around you in the right place and you have to put them in the right place … to do that, you do have to be able to speak to them… (P19, HPC‐F)

### 3.3 Health‐promoting activities and outcomes

Challenge: parent carers lack free time and experience negative impacts of caring on their physical health

Health and wellbeing outcomes were often thought about and articulated in terms of health‐promoting activities. However, engaging in positive health and wellbeing behaviours was difficult for some parent carers because of time constraints. Spare time was limited because ‘straightforward’ activities were often more complicated because of a child's needs. However, for some parents, feelings of guilt made them reluctant to take time for themselves even when they could find some time to spare.

Aspects of physical health were also discussed, and parent carers shared examples of how caring negatively impacted their physical health. Some participants described musculoskeletal symptoms which worsened due to their caring responsibilities—especially if their child had a physical disability.

I've got two discs out of my back from the constant lifting… I've constantly got a bad back … and there's a chance that I might need an operation. (P25, HPC‐NP)

All interviewees described feeling physically exhausted because of their caring responsibilities. Their lack of sleep was especially challenging and had a physical impact, but also a mental health impact, which included experiencing negative emotions more readily.

Physical aspects of health were perceived to be influenced by mental wellbeing. For example, parent carers described how their immune system was compromised by stress, and how their anxiety resulted in physical symptoms.

She wasn't getting special care at school … but I had to leave her there… I'd get home and I'd physically have to lie down because the anxiety was so overwhelming that I couldn't stand up. (P29, HPC‐NP)

Concerns about future physical health also created anxiety for some parent carers, including one older parent carer who worried about what her health and mortality meant for the future care of her child.

Change: engaging in health‐promoting activities

In terms of positive changes, parent carers described virtuous cycles where health‐promoting activities helped improve their health and wellbeing. For example, health‐promoting activities increased parent carers' energy levels, aided relaxation, and reduced stress). These feelings promoted further engagement with healthy activities. Parent carers who engaged in healthy behaviours also began to focus more on diet, exercise and sleep. Improving sleep was significant to parent carers because of how it affected various areas of health and wellbeing, including parents' ability to think more clearly and problem‐solve. For example, parents tried to protect their sleep by ‘winding down’—reducing phone use, reading or listening to relaxing sounds before sleep.

it's about switching your gadgets off or just having a nice long soak in the bath and that kind of thing. Yes, it does help. It gets you into a different pattern. (P1, HPC‐P)

Parent carers often equated increasing fitness with physical activity and gaining a healthy weight. However, some male parent carers, aware of the physical demands of caring for their children with physical disabilities, emphasised the importance of maintaining good physical condition for this specific reason.

I incorporate strength training and things like that … Not just because it's good the older you get but also because of knowing that there are times that you are having to do lifting… (P30, HPC‐NP)

Some parent carers highlighted the role of self‐knowledge when managing one's physical health. They explained that they needed to manage external stressors because these stressors are the trigger for their unhealthy behaviours. Health‐promoting behaviours combined with self‐knowledge helped these parents protect their health and wellbeing.

If I am finding things stressful in the moment, taking a step back mentally and having a moment to look at where I am at from a different perspective … really helps me to navigate a bit better. (P13, HPC‐P)

A final challenge described by interviewees was the difficulty sustaining behaviour change once an intervention had concluded. However, some former HPC participants successfully addressed this by revisiting their resources, creating new healthy practices and routines, and remaining connected to their peer group for ongoing encouragement.

## 4 DISCUSSION

This study explored which aspects of health and wellbeing are most meaningful to parent carers. Interviewees described some of the common stressors which contribute to the negative aspects of their health and wellbeing. We found that stressors result in parent carers' physical and psychological needs going unmet, and that this can result in a negative perspective on the ‘parent carer’ social identity. We also found that parent carers demonstrate a more positive view of the parent carer identity when their needs are met, which can result from participation in a peer‐led group‐based intervention. Our findings highlight that ‘needs‐based’ theories and social identity theories are important considerations when determining which aspects of health and wellbeing are most important to parent carers.

In terms of physical health, research has shown that parent carers are at greater risk of negative physical health outcomes, such as headaches and musculoskeletal pain.^38^ Our study supports these findings, adding that certain physical health outcomes are important to parent carers, such as becoming more physically fit, obtaining a healthy weight, gaining more energy, feeling fewer physical symptoms of stress and feeling more relaxed. While parent carers have previously highlighted the importance of sleep,^39^ our study found that sleep is especially important because it can mediate other outcomes, such as coping with psychological stressors and promoting mental health.

The link between being a parent carer and experiencing negative mental health outcomes is well established.^4^, ^5^, ^6^, ^7^, ^12^ For proponents of the ‘stress‐health’ model, the negative outcomes experienced by parent carers result from stressors common to this group, including the assessment and diagnosis journey; the day‐to‐day demands of the caregiver role and its impact on employment; and experiencing disability stigma.^40^ Our findings add to some of the emotional, cognitive and behavioural consequences of these stressors. For example, feelings of anxiety and uncertainty were frequently associated with the thought that, ‘I am not doing a “good enough” job’ as a parent carer. Being a ‘good enough’ parent carer usually meant investing most of their time and energy into meeting their child's needs—regardless of the cost to their own personal health and wellbeing. As such, our findings provide an example of what has been called the ‘Good Mothering’ ideology^41^; demonstrating how this ideology can impact parent carers' perception of their own identity.

Difficult emotions also promoted ‘emotion‐focused’ coping behaviours, such as avoidance, which can result in depressive symptoms, negative affect, fear, hostility and sadness.^42^ Interviewees described avoidance behaviours related to social stigma. For example, when friends, family, professionals or the public could not empathise with the parent carer experience—parent carers felt socially embarrassed and judged. These emotions motivated parent carers to withdraw from social interactions altogether, which led to reduced confidence and increased helplessness.

Overall, negative mental health outcomes and depressive symptoms are commonly reported among caregivers.^4^, ^43^ In our study, we also found that these outcomes were the result of a ‘downward spiral’ of thoughts, feelings and behaviours interacting with the common stressors outlined above. This ‘downward spiral’ can be understood in terms of the difficulty parent carers experience having their physiological and psychological needs met. For example, as a result of being awake to meet their child's needs throughout the night, parent carers' physiological need for sleep is not met.^44^

Parent carers also described how their competence, autonomy and relatedness needs are not met. In terms of a ‘downward spiral’, research has shown that negative competence self‐perceptions, autonomy frustration and social isolation have each been associated with decreased health and wellbeing outcomes,^45^, ^46^, ^47^, ^48^, ^49^ including stress and ‘burnout’.^46^, ^50^ Self‐determination may therefore be an appropriate model through which to identify the health and wellbeing outcomes most meaningful to parent carers. Self‐determination theory (SDT) should therefore be considered by researchers interested in measuring health and wellbeing outcomes among this group. It has also been argued in the literature that there is little existing consensus over development of a universal health and wellbeing self‐report tool, owing partly to conceptual ambiguity and disagreement of definition.^27^ Our findings suggest that self‐determination may play a role in shaping parent carers’ health and wellbeing priorities.

Parent carers' competence self‐perceptions, for example, are decreased by social comparisons.^47^ While positive feedback can help overcome this,^51^ socially isolated parent carers' do not receive relevant peer feedback, and so their negative self‐evaluations go unchallenged. Outcomes related to competence and belonging might therefore focus on whether parents receive feedback on their parenting which is ‘fair’, or whether they can discuss their problems with someone who ‘gets it’. This highlights the importance of connecting with relevant parent carer peers, as peer comparisons promote the ability to ‘sense check’ and normalise difficult experiences.

Decreased autonomy has also been linked to negative health outcomes,^52^ and parent carers often described feeling less autonomous, especially when it came to feeling a sense of control over their lives. Our findings showed that when parent carers were better able to accept what they could and could not change, this increased wellbeing.

Our findings also demonstrate that another important aspect of parent carer autonomy related to self‐care. Parent carers often described needing ‘permission’ to prioritise their own needs because they so often organised their lives around the needs of their children. Parent carers who were supported to put their needs first described experiencing more positive health and wellbeing outcomes. Outcomes related to autonomy might therefore focus on both self‐care.

The interviewees in our study who had more of their competence and autonomy needs met were most often those who had participated in the HPC programme. By participating in the programme, they were able to make more accurate social comparisons and receive empathetic peer feedback—increasing their competence self‐perceptions. Outcomes related to belongingness might therefore measure the extent to which parent carers feel stigmatised, judged or embarrassed in their social interactions.

The HPC programme helped parent carers meet their belongingness needs because it required them to take part in a group‐based, peer‐led intervention. While completing the course, parent carers were less isolated. However, previous research has shown that group‐based interventions do not simply meet group members' individual psychological needs. Instead, social identification processes facilitate the emergence of a shared social identity within the group and the positive attributes associated with this identity can then be internalised by group members, increasing self‐esteem and confidence.^53^, ^54^

In the case of HPC, the intervention promoted the transformation of the parent carer identity itself. Before the course, the parent carer identity was characterised by stressors common to this group, and an overwhelming sense of feeling ‘stuck in a rut’. After the course, the parent carer identity demonstrated many more positive associations—offering a sense of meaning and purpose, as well as a reduction in low mood, an increase in positive affect and an increased enjoyment of life. When thinking about measuring health and wellbeing outcomes among parent carers, it could be informative to think about the parent carer identity, asking questions about how parent carers feel about their parent carer identity, whether it is characterised in a positive or negative way and whether it dominates other aspects of an individual's identity. Questions might therefore focus on whether parent carers have opportunities to engage in hobbies and interests, and/or whether they perceive they are likely to explore their personal interests in the future.

### 4.1 Strengths and limitations

Our study presents robust thematic analysis, based upon consensus between multiple researchers and triangulation with other parent carers through the study PPI group. We also compared and contrasted our findings in terms of HPC facilitators, HPC participants and HPC nonparticipants, finding that they were consistent across these groups. The findings are therefore likely to have resonance and transferability to a variety of groups working in this field.

While efforts were made to recruit a diverse group of participants, two‐thirds of our participants were parent carers of children with autism. We therefore recruited more parents of children with physical disabilities to answer the research question more comprehensively. Four‐fifths of our participants were also white British, meaning that the nuanced ways in which ethnic‐cultural characteristics influence attitudes and beliefs about health and wellbeing are less prevalent in our findings. Nevertheless, the four parent carers in our sample from a British Asian background outlined that—while differences might exist in perspectives around health and wellbeing in their community—the core challenges facing parent carers were common across each ethnic group.

### 4.2 Implications and future research

Researchers are encouraged to seek the most relevant areas of wellbeing for their population of interest, and work is still needed to accurately describe the precise outcomes which should be focused on when measuring parent carers' health and wellbeing—particularly when evaluating parent carer‐focused interventions. However, this study has demonstrated that parent carers often understand health and wellbeing in terms of the common challenges they face as a group. It has also highlighted the challenges faced by this group when seeking to have their physical and psychological needs met—which can result in a negative view of themselves as parent carers. Like previous studies which have focused on activities,^17^ or capabilities,^55^ our findings suggest that health and wellbeing outcomes among parent carers might be better measured with reference not only to specific outcome domains and broader conceptual frameworks, such as SDT and social identity theory.^54^ A ‘context‐specific’ measure that frames wellbeing outcomes in terms of parent carers’ experiences,^27^, ^56^ and outcomes based on broader conceptual frameworks,^17^, ^55^ is a potential next step.

## AUTHOR CONTRIBUTIONS

**Caomhan McGlinchey**: Writing—original draft; writing—review and editing; formal analysis; investigation; conceptualisation; methodology. **Phillip Harniess**: Conceptualisation; investigation; writing—original draft; writing—review and editing; methodology; formal analysis. **Aleksandra J. Borek**: Conceptualisation; funding acquisition; writing—review and editing; writing—original draft; methodology; project administration; supervision. **Alice Garrood**: Conceptualisation; funding acquisition; writing—review and editing. **Annabel McDonald**: Conceptualisation; funding acquisition; writing—review and editing; project administration. **Fleur Boyle**: Funding acquisition; writing—review and editing; project administration; resources. **Stuart Logan**: Conceptualisation; funding acquisition; methodology; writing—review and editing; supervision. **Christopher Morris**: Conceptualisation; funding acquisition; writing—original draft; writing—review and editing; project administration; resources; supervision; methodology.

## CONFLICT OF INTEREST STATEMENT

The authors declare no conflict of interest.

## ETHICS STATEMENT

The study procedures were approved by the University of Exeter Medical School Research Ethics Committee (UEMS REC ID: 525009).

## Data Availability

The data that support the findings of this study are available on request from the corresponding author. The data are not publicly available due to privacy or ethical restrictions.
